# Intravesical gemcitabine as bladder‐preserving treatment for BCG unresponsive non‐muscle‐invasive bladder cancer. Results from a single‐arm, open‐label study

**DOI:** 10.1002/bco2.28

**Published:** 2020-07-01

**Authors:** Rodolfo Hurle, Paolo Casale, Emanuela Morenghi, Alberto Saita, Nicolòmaria Buffi, Giovanni Lughezzani, Piergiuseppe Colombo, Roberto Contieri, Nicola Frego, Giorgio Guazzoni, Massimo Lazzeri

**Affiliations:** ^1^ Department of Urology Humanitas Clinical and Research Center - IRCCS Rozzano Italy; ^2^ Department of Medical Statistic Humanitas Clinical and Research Center - IRCCS Rozzano Italy; ^3^ Department of Biomedical Sciences Humanitas University Milan Italy; ^4^ Department of Pathology Humanitas Clinical and Research Center - IRCCS Rozzano Italy

**Keywords:** bladder cancer, BCG, gemcitabine, intravesical therapy

## Abstract

**Background:**

There is an unmet alternative medical therapy for BCG unresponsive patients.

**Objective:**

To report efficacy of intravesical gemcitabine in NMIBC patients, who failed a previous course of BCG, or intolerant, and unwilling to undergo radical cystectomy (RC).

**Material and methods:**

This is an open‐label, single‐arm study, which enrolled patients showing a failure or were intolerant to BCG and unwilling to undergo the RC. Intravesical gemcitabine was administered once a week for six consecutive weeks and once a month for 12 months. The primary outcome was DFS defined as the lack of a tumor on cystoscopy and negative urine cytology. Secondary endpoint was safety defined according a grading of side effects. OS, PFS, and DFS were described with Kaplan–Meier method at 12 and 24 months.

**Results and limitations:**

Overall 36 patients were enrolled. The median follow‐up was 27 months. The DFS was 68.75% at the end of induction phase and 44.44% and 31.66% at 12 and 24 months of, respectively. The PFS was 43.75%. The OS and CSS were 77.9% (95% CI 58.78%‐88.92%) and 80.68% (95% CI 61.49%‐90.96%), respectively. There was no life threatening event or treatment‐related death (grade 4 or 5). The most common mild and moderate adverse events reported were urinary symptoms (LUTS) and fatigue (G1‐G2).

**Conclusion:**

Patients who presented an unresponsive‐BCG recurrent NMIBC and unwilling to receive a RC, could benefit from intravesical gemcitabine as salvage organ‐sparing treatment.

## 1 INTRODUCTION

Bladder cancer (BC) is a common neoplasm of the urinary tract. It is the ninth most common malignancy worldwide and the fourth most common cancer in males being nearly three/four times more common in men compared in women.^1^ The disease remains confined to the layers above the muscularis propria in approximately 75%‐85% of patients, and it is defined as non‐muscle‐invasive BC (NMIBC). High grade NMIBC and *carcinoma* in situ (CIS) are high‐risk conditions for progression to muscle invasive BC (MIBC). In the absence of any adjuvant treatment, up to the 90% may recur after transurethral resection (TURBT) and about half of patients’ progress to MIBC.^2^ The recommended treatment of high risk NMIBC is a six weekly induction course with intravesical instillations of bacillus Calmette‐Guérin (BCG) followed by maintenance courses.^3^ BCG fails in up to 50% of patients and it may be associated with local or systemic adverse events in approximately 70% of them.^4^ Approximately 5% to 9% of patients abandon the treatment due to adverse events not completing all the planned BCG courses.^4^ Recently supplies of BCG continued to dwindle leading to a world‐wide shortage, which limits the access to the treatment.^5^

Radical cystectomy (RC) is the recommended standard‐of‐care treatment for BCG unresponsive or intolerant patients, although a relevant morbidity is reported, regardless of “open” vs “robotic” approaches.^6^, ^7^ Furthermore a substantial proportion of patients are either unfit for or unwilling to undergo surgery. Thus, there is a rising need for bladder‐sparing alternatives. In the last decade several new agents, which can be delivered more safely and efficiently to the bladder, have been investigating.^3^, ^8^ Unfortunately, development of new drugs in this disease space has been hampered by the heterogeneity in patient population, poor definition of disease status, uncontrolled studies, and consensus on outcome definition. There is an unmet clinical need for effective bladder‐sparing agents to treat recurrent NMIBC not responsive to BCG.

Gemcitabine (2′,2′‐difluorodeoxycytidine) is a well‐known chemotherapeutic agent that inhibits DNA synthesis in dividing cells. Regimens containing gemcitabine are used systemically to treat MIBC and advanced urothelial cancer.^9^, ^10^ Sporadic evidence suggested that courses of intravesical gemcitabine could be safe and cost/effective for BCG unresponsive NMIBC, but most of those studies were limited by small number of patients and short‐term follow‐up.^11^, ^12^ Here, we report our experience with intravesical gemcitabine in NMIBC patients, who failed a previous course of BCG and unwilling to undergo RC.

## 2 MATERIAL AND METHODS

This is an open‐label, single‐arm study, approved by our Institutional Review Board and local Ethical Committee (n.1313_codice_ICH‐007_V1.00), which enrolled patients showing a failure or were intolerant to BCG and unwilling to undergo the recommended standard‐of‐care RC. Patients were deemed to be unresponsive to BCG if they presented a recurrent NMIBC (confirmed CIS, pT1, or pTa multifocal high grade) 3 months after an induction cycle or during the maintenance treatment. BCG intolerant patients were considered all them who dropped out due to a serious adverse event requiring the discontinuation of BCG therapy.^13^

All patients were informed about the disease course after BCG failure, guideline indications, and potential bladder‐preserving treatments. Patients, who refused RC, were offered a salvage intravesical gemcitabine therapy. After a shared and informed discussion, they signed the informed consent. Exclusion criteria were: incomplete pathological data, severe cardiovascular diseases within the last 6 months, MIBC, concomitant cancer of the upper urinary tract, previous immunotherapy or chemotherapy. Severe acute or chronic medical or psychiatric condition that might increase the risk of not attending the treatment and follow‐ups, and interfere with the interpretation of study results, were considered exclusion criteria.

Gemcitabine (2000 mg in 50 mL) was administered once a week for six consecutive weeks in the intensive phase. Patients were asked to retain the drug in the bladder for 120′. Patients who achieved a disease‐free survival (DFS), defined as the lack of a tumor on cystoscopy evaluation with mandate/for‐cause biopsy and negative urine cytology, entered the maintenance phase of the study, during which gemcitabine is further administered once a month for 12 months. The first monthly instillation occurred about 4 weeks after the end of the induction course.

The primary objectives were: antitumor efficacy of gemcitabine defined as DFS, ≥T2/T4 or extravesical disease progression‐free survival (PFS) and cancer‐specific survival (CSS). The first follow‐up was performed 3 weeks after the end of induction phase, then cystoscopy and urine cytology were performed every 3 months. At the end of maintenance course a bladder mapping was scheduled. The secondary objective was to evaluate the safety and tolerability profile of gemcitabine given by intravesical instillation for six consecutive weeks and for 12 months in patients who obtained a DFS at the end of induction. Overall safety profile was evaluated on the basis of laboratory and clinical safety parameters (ie, hematology and blood chemistry, urinalysis, vital signs, and adverse events emerging during the trial: severe lower urinary tract symptoms—LUTS, hematuria, fever, and pelvic pain). The NCI Common Terminology Criteria for Adverse Events CTCAE Version 4.03 has been used for the severity grading of adverse events.^14^

### 2.1 Statistical analysis

Data were described as numbers and percentages, if categorical, or mean and standard deviation, if continuous. Overall Survival (OS) time was calculated from the date of TURB to the date of death or last contact date. PFS time and DFS time were calculated from the date of TURB to first progression or recurrence date, respectively, or last contact date. OS, PFS, and DFS were described with Kaplan–Meier method. Survival at 12 and 24 months were also described as percentage and 95% confidence interval (CI). All analyses were made with Stata 15.

## 3 RESULTS

From February 2012 to October 2018, 36 patients were included in the study. Demography of enrolled patients is reported in Table 1. All patients were White Caucasians with a mean age of 70.25 ± 7.69, and a ratio male/female of 29/7. Over 32 patients, who completed the 6‐week induction BCG course, 10 presented a failure with a DFS of 68.75% at the end of induction phase. Twenty‐two patients showed a relapsing during their maintenance phase. Four patients were intolerant and did not complete the induction phase. The overall number of instillation was 344 with 9.5 mean instillations per patient. Seven patients had received MMC previously to BCG.

**Table 1:** Demographic and clinical characteristics of patients

*N*	36
Gender (M)	29 (80.56%)
Age	70.25 ± 7.69
BMI	24.32 ± 2.69
Physical activity	30 (83.33%)
Hypertension	20 (55.56%)
Diabetes	19 (52.78%)
Smoking status	
Never	10 (27.78%)
Former	5 (13.89%)
Actual	21 (58.33%)
Pathological features	
HG multifocal pTa	1 (2.8%)
pT1 (only)	18 (50%)
CIS + papillary disease	7 (19.4%)
CIS (only)	10 (27.78%)

**Figure 1:**
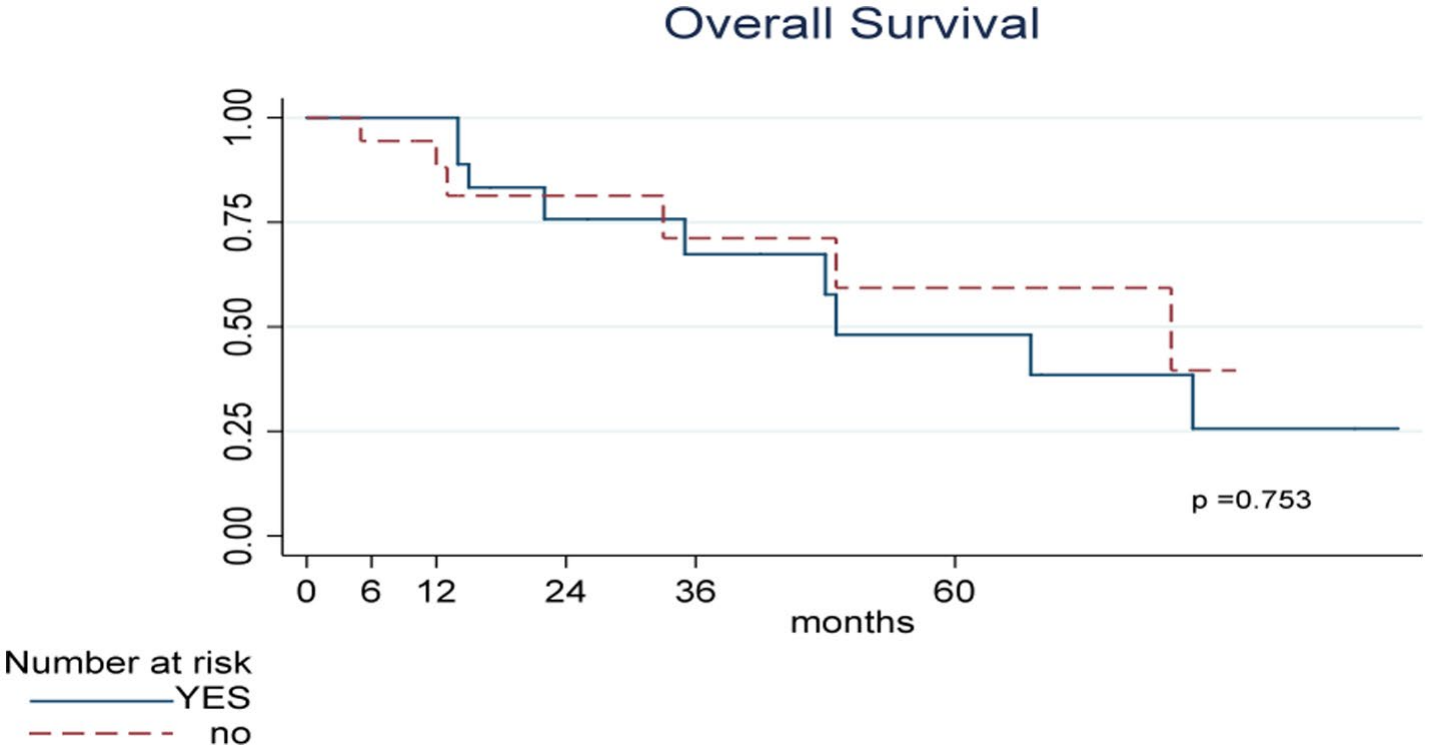
Kaplan–Meier curves of overall Survival (OS) stratified according to DFS after induction. OS time calculated from the date of TURB to the date of death or last contact date

**Figure 2:**
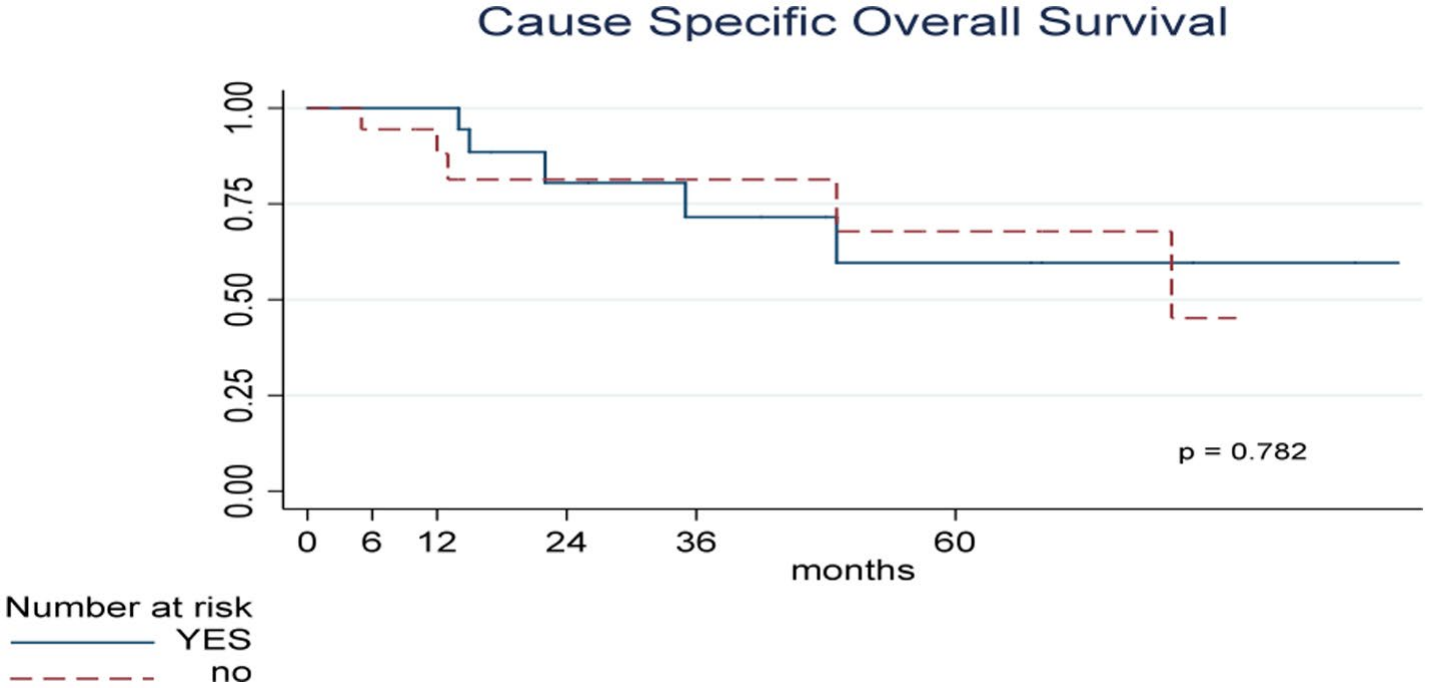
Kaplan–Meier curves of Cancer‐Specific Survival (CSS) stratified according to DFS after induction. CSS time calculated from the date of TURB to the date of death or last contact date

The median interval between the last BCG instillation and the beginning of intravesical gemcitabine was 2 months. The median follow‐up was 27 months (interquartile range 14.5‐62). The overall survival at 24 months was 77.9% (95% CI 58.78%‐88.92%) associated with a cancer‐specific survival (CSS) of 80.68% (95% CI 61.49%‐90.96%). Nine patients reached a 5‐year follow‐up with a CSS of 63.03% (95% CI 38.69%‐79.90%). Data are summarized in Figures 1 and 2. None OS or CSS was different for DFS after induction results (*P* = .753 and *P* = .782, respectively).

**Figure 3:**
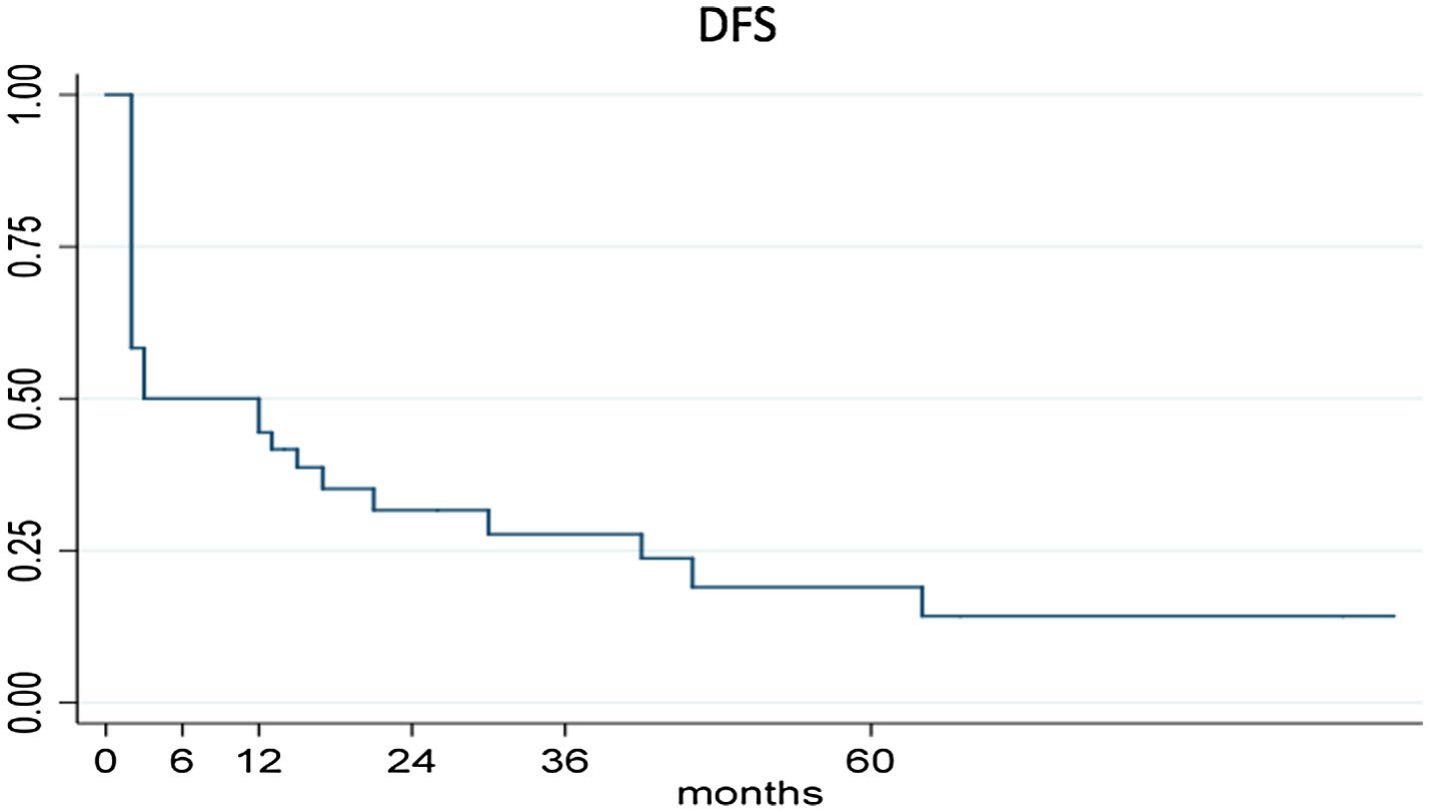
Kaplan–Meier curves of DFS. DFS time calculated from the date of TURB to first recurrence date, or last contact date

The DFS at 12 and 24 months for the whole sample was 44.44% (95% CI 28.02%‐59.64%) and 31.66% (95% CI 16.97%‐47.43%), respectively. Figure 3 showed the K–M curves for DFS. If we consider only patients with a DFS at the end of induction the DFS at 12 and 24 months was 88.89% (95% CI 62.42%‐97.10%) and 63.31% (95% CI 35.32%‐81.81%), respectively. Eleven patients presented a recurrence without any progression.

**Figure 4:**
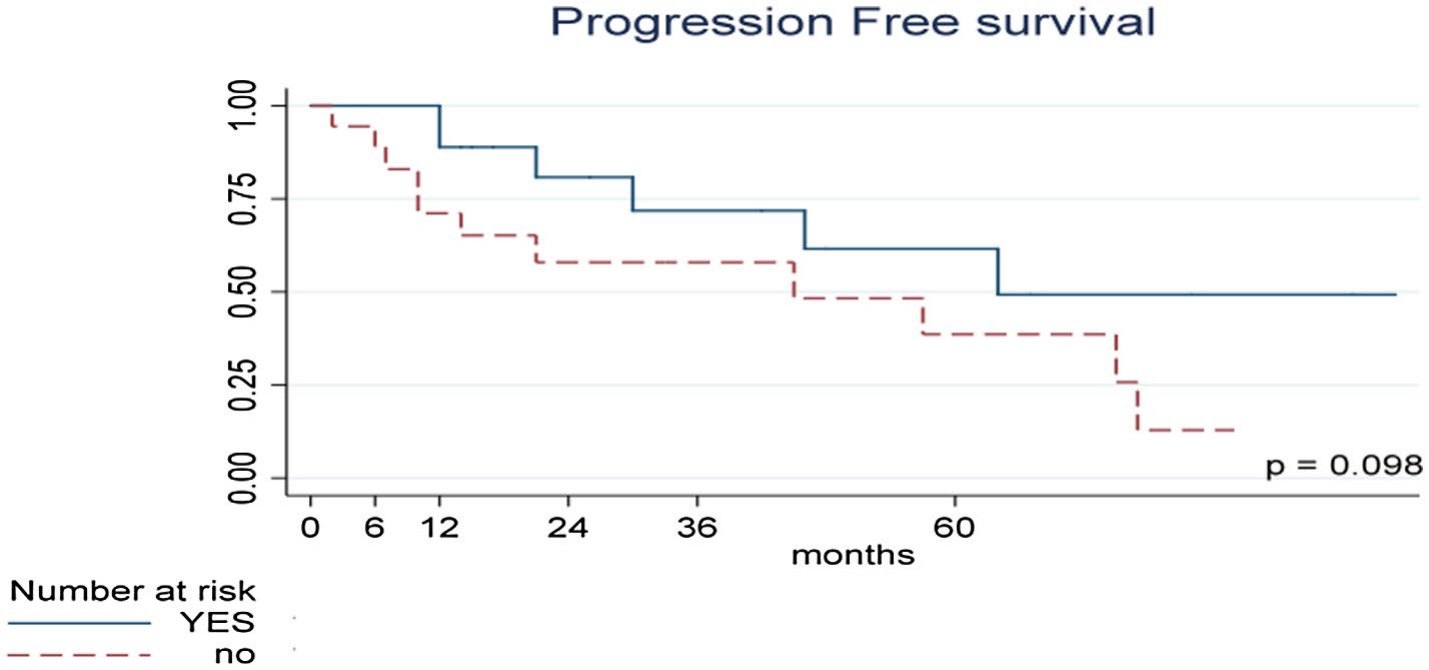
Kaplan–Meier curves of Progression Free Survival (PFS) stratified according to DFS after induction. PFS time calculated from the date of TURB to first progression, or last contact date

Overall 14 patients presented a progression to T2‐T4 or extravesical lesions. Progression free survival at 12 and 24 months was 80.13% (95% CI: 62.78%‐90.00%) and 69.55% (95% CI: 50.33%‐82.52%), respectively. Nine patients reached a 5‐year follow‐up with a PFS of 49.85% (95% CI 28.78%‐67.76). Figure 4 showed the K–M curve for PFS. We cannot find a strictly statistical difference when patients were stratified according to DFS or not at the end of induction (*P* = .098).

Patients generally tolerated intravesical gemcitabine well; Table 2 summarizes side effects due to the drug. According to CTCAE, there was no life threatening event or treatment‐related death (grade 4 or 5). The most common mild and moderate adverse events reported were urinary symptoms (LUTS) in 14 cases and fatigue in 12 (G1‐G2). Three patients experienced CTCAE grade 3: gross hematuria, severe bladder pain, and severe pelvic pain in one female subject that required discontinuation of the therapy.

**Table 2:** Side effects of intravescical gemcitabine

Side effects	No. Grade 1 or 2 (%)	No. Grade 3 (%)
Urinary	14	–
Fatigue	12	–
Hematuria	–	1
Fever < 38,5	6	–
Fever > 38,5	–	7
Pelvic pain	–	2

Ten patients who failed the gemcitabine salvage treatment underwent RC: one due to recurrent nonprogressive NMIBC and nine for progression to MIBC or extravesical lesions. Two patients who presented a progression to metastatic disease received chemotherapy and they did not undergo RC.

## 4 DISCUSSION

We found that salvage intravesical gemcitabine, for a selected unresponsive BCG population, achieved an overall DFS at 12 and 24 months of 44.44% and 31.66%, respectively. These results are superior to data recently reported in a systematic review, analyzing eight studies, using different agents and enrolling CIS and/or papillary patients (similar to our series), with a DFS of 29% at 12 month.^15^ At the median follow‐up of 27 months, our study showed 14 (43.75%) patients with progression, which is a PFS lower respect to what reported by Li et al with a PFS of 94%, in studies including both CIS and/or papillary disease, at a median follow‐up of 17 months.^15^

Although guidelines recommend RC for BCG failure, it remains is a surgical procedure with significant morbidity and mortality rates, as well as dramatic lifestyle changes.^16^ Given the risk of progression and the critical need to balance the safety of RC with bladder preservation, the treatment of those patients is a personal decision, better if it is made through a shared decision. Under such prospective, it appears that there may be a window of opportunity to explore second line salvage intravesical therapies. Several treatments, alternatives to RC have being tested in patients with persistent or recurrent NMIBC or CIS after BCG therapy, but unfortunately, there exists no data‐driven efficacy benchmark for salvage bladder‐sparing therapy.^15^ Food and Drug Administration has approved valrubicin as the only agents for BCG unresponsive patients, but clinical results were disappointed with a complete response rate of 13% after 12 months.^17^

Gemcitabine‐based chemotherapy has been used for treating MIBC^9^, ^10^ and intravesically for high risk NMIBC. Porena et al carried out a RCT vs BCG in 64 patients with high‐risk NMIBC, including patients with CIS.^12^ They found that tolerability was better for gemcitabine, but it was less effective than BCG. At a mean follow‐up of 44 months, the recurrence rate in patients treated with BCG was 28.1% vs 53.1% of gemcitabine group (*P* = .037) without any disease progression. Authors concluded that gemcitabine is significantly inferior to BCG, but its favorable toxicity profile makes it useful for patients intolerant to BCG. Such data were not confirmed in a recent meta‐analysis comparing the efficacy and safety of intravesical gemcitabine with BCG in 386 NMIBC patients.^18^ Authors showed that there was no statistically significant difference in recurrence risk between intravesical gemcitabine vs BCG and no difference in progression risk as well. They confirmed that intravesical gemcitabine therapy had significantly lower incidence of dysuria and hematuria compared to BCG. The SWOG S0337 randomized clinical trial investigated the efficacy of a single intravesical instillation of gemcitabine immediately after TURBT to prevent the recurrence of low‐grade pTa or pT1 urothelial cancer of the bladder.^19^ Of the 201 patients randomized to receive gemcitabine (gemcitabine 2 g in 100 mL of saline vs saline) and 205 to receive saline in the intention‐to‐treat analysis, 35% vs 47%, respectively, experienced a recurrence by 4‐year median follow‐up (HR, 0.66; 95% CI, 0.48‐0.90; *P* < .001). Gemcitabine was administered in combination with other drugs, like everolimus or docetaxel, in patients after BCG failure.^20^ Steinberg et al investigated 276 BCG failure patients who received intravesical gemcitabine plus docetaxel.^21^ They reported 1 and 2‐year recurrence‐free survival (RFS) of 60% and 46% with only 10 patients (10/276, 3.6%) who had disease progression. Forty‐three (43/276, 15.6%) patients went on to RC (median 11.3 months from induction), of which 11 (11/276, 4.0%) had progressed to MIBC. Gemcitabine plus MMC has also been investigated with an initial report of 50% DFS at 18 months follow‐up.^22^

A SWOG study evaluated, as single‐agent, intravesical gemcitabine and found RFS rates of 28% at 1 year and 21% at 2 years post‐therapy.^23^ Notably, this study was conducted in patients with two previous BCG failures and utilized a 6 weeks induction course, followed by monthly maintenance for 12 months. Sternberg et al used two courses of intravesical gemcitabine (2000 mg instilled in 100 mL saline) twice weekly for 3 weeks with courses separated by a week of rest for a total of 12 instillations.^24^ After gemcitabine treatment, 27 patients had complete response (CR), 19 had partial response, and 20 had failure. The 5‐year cumulative incidence of death from bladder cancer was 12% and 18% in patients with and without a CR, respectively. In most of those studies, the definition of BCG‐unresponsive NMIBC was not homogeneous and they used interchangeably different outcome measures as complete response rate (CRR), recurrent‐free rate (RFR), or disease‐free rate (DFR), making comparisons with our data difficult (Table 3).

**Table 3:** DFS recorded at the first follow‐up after the induction phase

Author	Drug	No. patients (overall)	Trial design	DFS at the first F‐U
Skinner EC	Gemcitabine 2000 mg	47	Open‐label, single arm (Phase II)	22/47 (47%)
Sternberg IA	Gemcitabine 2000 mg	69	Retrospective	27/69 (39%)
Hurle R. et al (current data)	Gemcitabine 2000 mg	36	Open‐label, single arm	22/32^a^ (68.75%)

^a^ Four patients did not completed the induction course.

According to the international Bladder Cancer Group, CRR should be reserved for studies limited to CIS patients, RFR for patients with papillary disease and DFR for studies enrolling a combination of CIS and papillary cancer.^25^ Our population include patients with pure CIS, papillary disease and combination of both, and we used DFS as outcome measure having the words “rate” and “survival” the same purpose. When the outcome was stratified according the DFS at the first follow‐up, the DFS at 12 and 24 months was 88.89% (95% CI 62.42%‐97.10%) and 63.31% (95% CI 35.32%‐81.81%), respectively. An expert panel, supported by FDA and American Urological Association, stated that an initial response rate of at least 40%‐50% at 6 months and a durable response rate at least 30% for 18‐24 months could be considered clinically meaningful.^26^ Our study demonstrated to achieve a DFS of 50%, 44.44%, and 31.66% at 6, 12, and 24 months, respectively, making intravesical gemcitabine very interesting as salvage, bladder‐sparing therapy in BCG unresponsive NMIBC.

The current study presents not negligible weaknesses. Our population included patients with pure CIS, pure papillary disease, and combination of both and it should be tested not with an open‐label, single‐arm clinical trial, but by a RCT as stated by FDA’s Guidance Document on new therapies for NMIBC.^26^ The document was published in 2014, while we designed the study in 2012, and it explains the deviation from FDA guideline. The sample size remains small: it consists of 36 patients who were enrolled and only 32 who ended the induction course. However, in the systematic review reported by Li et al, over a total of 42 studies, consisting of 2254 patients, they presented a median of 35 patients per study (interquartile range 18‐47), which makes our series not negligible.^15^ At the beginning of our study, we did not use enhanced‐cystoscopy, and it could represent a limitation in accuracy of detecting all recurrent NMIBC. Although the use of enhanced cystoscopy has been demonstrated to improve detection rate of NMIBC, the effect of its adoption in the assessment of treatment efficacy of bladder‐sparing drugs in BCG unresponsive settings remain unclear.^15^, ^27^ It was reported that patient with papillary‐only BCG unresponsive NMIBC are more effectively treated with bladder‐sparing therapy than patients with pure CIS.^15^ Unfortunately, we did not perform any sub‐analysis of those populations. Furthermore, we did not use any stratification of risk by using biomarkers or molecular characterization.^28^ Yang et al investigated the predictive role of Ribonucleotide reductase subunit M1 (RRM1) mRNA in patients who received gemcitabine.^29^ A total of 1000 mg of gemcitabine was diluted in 40 mL of saline solution, and patients received weekly instillations for eight consecutive weeks and once a month for 1 year. A low RRM1 expression was associated with longer progression‐free survival and lower 1‐year/2‐year relapse rates in NMIBC patients treated with intravesical gemcitabine monotherapy. Finally, no assessment of quality of life was performed in our population, although it might represent an important outcome.^30^

## 5 CONCLUSION

Intravesical gemcitabine seems to represent a useful treatment for patients unresponsive or intolerant to BCG, unwilling to undergo the recommended standard‐of‐care RC. In order to support the evidence we reported, randomized clinical trials, with larger sample size and extended follow‐up, remain mandatory to determine the optimal gemcitabine regimen.

## CONFLICT OF INTEREST

The authors have no conflict of interest to report.

## AUTHOR CONTRIBUTIONS

Conception: Rodolfo Hurle; Performance of work: Roberto Contieri, Nicola Frego; Analysis of data: Emanuela Morenghi; Writing the article: Massimo Lazzeri.
